# The Significance of Matrix Metalloproteinase 9 (MMP-9) and Metalloproteinase 2 (MMP-2) in Urinary Bladder Cancer

**DOI:** 10.3390/biomedicines11030956

**Published:** 2023-03-20

**Authors:** Jacek Kudelski, Anna Tokarzewicz, Monika Gudowska-Sawczuk, Barbara Mroczko, Piotr Chłosta, Marta Bruczko-Goralewska, Przemysław Mitura, Grzegorz Młynarczyk

**Affiliations:** 1 Department of Urology, Medical University of Bialystok, M. Skłodowskiej-Curie 24A St., 15-276 Białystok, Poland; 2 Department of Medical Biochemistry, Medical University of Białystok, Adama Mickiewicza 2C St., 15-089 Białystok, Poland; 3 Department of Biochemical Diagnostics, Medical University of Białystok, Waszyngtona 15A St., 15-269 Białystok, Poland; 4 Department of Neurodegeneration Diagnostics, Medical University of Białystok, Waszyngtona 15A St., 15-269 Białystok, Poland; 5 Department of Urology, Jagiellonian University Medical College, Jakubowskiego 2 St., 30-688 Kraków, Poland; 6 Department of Urology, Medical University of Vienna, Währinger Gürtel 18-20 St., 1090 Vienna, Austria; 7 Department of Urology and Oncological Urology, Medical University of Lublin, Jaczewskiego 8, 20-954 Lublin, Poland

**Keywords:** urinary bladder carcinoma, gelatinase, MMP-2, MMP-9, biomarker

## Abstract

Introduction: Urinary bladder cancer is a serious oncological problem that is the cause of many deaths worldwide. The processes of metastasis and origination of local tumor invasion depend on the extracellular matrix (ECM) degradation. The cancer microenvironment, particularly the ECM, may be considered a key factor in cancer progression. Matrix metalloproteinases (MMPs) are classified as the main factors responsible for the degradation of ECM components. Therefore, the aim of the study was to evaluate the expression and activity of matrix metalloproteinase 2 and 9 (MMP-2 and MMP-9) in urinary bladder cancer according to different stages. Material and methods: Urinary bladder tissue samples were analyzed. Cancer patients were divided into two groups: low-grade tumors (LG; Group I) and high-grade tumors (HG; Group II). Control tissue was obtained from the opposite site to the tumor. MMPs content and activity (actual and specific) were evaluated using ELISA and Western blot methods, respectively. Results: Both MMPs are present in high and low molecular complexes in healthy or bladder cancer tissues. The content of MMP-9 is enhanced in comparison with MMP-2, particularly in HG cancer tissue. The actual activity of MMP-2 was highest in LG cancer tissue whereas the actual activity of MMP-9 was highest in HG cancer. Specific activity of both MMPs was highest in LG cancer, but the activity of MMP-9 was higher in comparison with MMP-2. Conclusions: In conclusion, the content and specific activity of MMP-9 were increased in comparison with MMP-2. The revealed differences in content and activity of both MMPs demonstrate their different participation in ECM remodeling at different stages of cancer development. Moreover, it seems that MMP-9 has higher clinical utility than MMP-2 as a potential therapeutic option and a diagnostic biomarker of urinary bladder cancer.

## 1. Introduction

In the developed countries, urothelial carcinomas (UCs) are the fourth most common tumors [1]. They can be located in the two parts of the urinary tract: in the upper (pyelocaliceal cavities and ureter) and/or in the lower (bladder and urethra) part.

The urinary bladder must resist the highly dangerous environment of urine. Any pathological condition of urinary bladder beginning with an inflammation and resulting in a cancer, leads to its structural and functional damages. Therefore, such problems as dysuria or hematuria may occur in the course of numerous diseases, such as bladder cancer (BCa), which is the most common type of urinary tract malignancy accounting for approximately 90–95% of all urothelial carcinoma cases [2]. The factors which increase the risk of BCa developing are age and gender. It was found that the risk is almost 3–4 higher in men than in women [3]. BCa frequently results in patients’ deaths [4,5,6]. That is why the search for a new BCa markers is important for early diagnosis and prognosis what lead to increased chances of patients’ survival.

Extracellular matrix (ECM) and its properties control the functions of the urinary bladder. The ECM structure of the bladder is well known and described [7]. The collagen is one of the main ECM components of the urinary bladder and constitutes over 57 percent of dry weight of the entire insoluble proteins. A high collagen amount was discovered to be related to its extensibility function in the course of filling of the bladder lumen. The collagen degradation depends on such factors as the activity of the matrix metalloproteinases (MMPs). It was found that activities and/or expression of the two gelatinases MMPs—MMP-2 and MMP-9—are changed during the BCa development [3,8,9,10,11].

Matrix metalloproteinase 2 (MMP-2, gelatinase-A) has a molecular weight of 72 kDa whereas an active form of the enzyme weighs 66 kDa. Its main substrates are types I, II, III, IV, V, VII, X, XI of collagen. Apart from collagens, also other molecules are degraded by MMP-2: fibronectin, gelatin, proteoglycans, laminin, and aggrecan elastin. Known as a neutrophil gelatinase, it is involved in many different processes: vascular remodeling, angiogenesis, tissue repair, tumor invasion, and inflammation. It has been reported that MMP-2 is associated with many disorders such as: multicentric osteolysis, nodulosis, and arthropathy [12,13].

Matrix metalloproteinase 9 (MMP-9 or gelatinase-B) is involved in extracellular matrix remodeling in various pathological states. Gelatinase-B weighs 92 kDa whereas the active form weighs 86 kDa and is slightly heavier than MMP-2. MMP-9 main types of substrates are: fibronectin, elastin, gelatin, aggrecan, and types IV, V, VII, X, XIV of collagen [12].

Both gelatinases degrade pre-digested various types of collagen of which the most important seems to be type IV of collagen—a basic constituent of basement membranes.

It has been proven that the activity of MMP-2 in bladder cancer is markedly elevated in comparison with the healthy tissue. It also correlates with the degree of malignancy and aggressiveness of the tumor [14,15]. The gelatinase-A expression is regulated by the fibroblast growth factor which is known as a representative of cytokines. Considering the blood test results, healthy patients represented a similar MMP-2 concentration in comparison with patients with urinary bladder cancer [16]. It was shown that better clinical outcome was associated with high concentrations of circulating TIMP-2 and pro-MMP-2. It suggests that pro-MMP-2 may be taken into consideration as a bladder cancer marker. There is still a large area that should be taken under investigation for early cancer diagnosis [17,18,19].

Research on bladder cancer and stromal cells demonstrated the MMP-9 expression and increased activity [20,21]. However, no gelatinase-B expression in healthy epithelium was found [22]. It was found by Kader et al. [23] that overall and invasive bladder cancer risks were associated with genetic variations in MMP-9. No significant relation between MMP-9 and local invasion of urinary bladder tumor was demonstrated during other studies. However, a higher degree of tumor malignancy correlated with a higher MMP-9 concentration in blood [24]. What still remains disputable is the potential significance of determination of the concentration of MMP-2 and MMP-9 in urine as an important predictor of urinary bladder cancer progression [25,26].

There is a number of research studies concerning the activity and content of MMPs in urinary bladder cancer. Their outcomes often differ. Such divergences may be caused by the methodology and tissue material applied. Most of studies were performed in urine and blood samples of cancer patients. It seems that these results are not exactly sufficient for the real evaluation of metalloproteinases participation in abnormal tissue development. Therefore, with regard to the observation mentioned above, we tried to evaluate the content, activity, and expression of both MMP-2 and MMP-9 in neoplastic tissue of the bladder compared with the control tissue.

## 2. Materials and Methods

The Bioethical Committee of the Medical University of Bialystok gave permission to this study (RI002/220/2015; date of approval: 28 May 2015).

### 2.1. Tissue Material

The study concerned the main type of urinary bladder cancer—urothelial cancer—in two phases of morphological malignancy. All samples were taken during surgical procedures at the Department of Urology, Medical University of Bialystok. The patients underwent transurethral resection or radical cystectomy of a bladder tumor. The urothelial cancer was diagnosed using histopathological methods, which allowed us to divide patients into two study groups based on the aggressiveness of the cancer (grading). To the presented study were taken two groups of patients: diagnosed with a low-grade (LG) cancer (10 patients) and with a high-grade (HG) cancer (10 patients). Firstly, the urinary bladder was removed during open surgery. Secondly, the samples were collected from the areas of macroscopically visible cancer tissue. After a radical open cystectomy procedure, control tissue was obtained from each patient from the area opposite to the tumor. However, collecting non-cancerous tissues during the transurethral resection of the bladder was not possible.

Range of the patients’ age was 47–91 years old and its average was 70.3 years old. The ratio of women to men in each study group was 2:1.

Tissue extracts were prepared according to the method described in the manuscript by Bruczko, M. et al. [27] using a special buffer without any additional compounds.

### 2.2. Overall Collagenolytic Activity Evaluation

Gelatin was used as a substrate for degradation to evaluate the collagenolytic ability of investigated gelatinases. Their activity in all investigated tissues was evaluated with the zymographic technique [28]. Tissue extracts (20 µg of protein) were exposed to electrophoresis with the use of a 10% polyacrylamide gel containing gelatin with a concentration of 1.5 mg/mL of gel. Electrophoresis was conducted at a constant voltage of 150 V.

The gels were then submitted to extraction of sodium dodecyl sulphate (SDS) using 2% Triton X-100, at 37 °C for 1 h. The gels were transferred into Tris/HCl (0.05 mol/L) in pH 8.0, containing 5 mM CaCl_2_, and incubated at 37 °C for 18 h. In the next step, the gels were stained with 1% Coomassie Brilliant Blue. Each gel was colored dark blue despite regions where gelatin degradation took place. Representative zymogram was demonstrated.

### 2.3. Gelatinases’ Content

The content of gelatinase A in the examined materials was determined using Quantikine ELISA Total MMP-2 Immunoassay (Cat#MMP200) and the gelatinase B content with Quantikine ELISA Human MMP-9 Immunoassay (Cat#DMP900) (both provided by R&D systems, Minneapolis, MN, USA) according to the manufacturer instructions.

### 2.4. Gelatinases’ Western Blot

According to the Laemmli method, all samples (10 μg of protein) were electrophoresed on SDS-polyacrylamide gel (10%) [29], blotted to nitrocellulose membranes (Sigma-Aldrich; Saint Louis, MO, USA) at 100 mA for 1 h. The membranes were blocked using 5% (*w*/*v*) nonfat powdered milk in the solution of TBS-T (20 mM Tris/HCl buffer, pH 7.4, 150 mM NaCl, 0.05% (*v*/*v*) Tween 20) for 1 h. Then, samples were incubated overnight at 4 °C with antibodies against metalloproteinase-2 (Cat#MAB9021; R&D Systems, USA) or, respectively, directed against metalloproteinase-9 (Cat# MAB936; R&D Systems; USA) in TBS-T which contained 1% bovine serum albumin (*w*/*v*). In the next step, several washes in TBS-T buffer were performed. Bounded antibodies were detected using alkaline phosphatase (ALP) coupled with the appropriate antibody in the same solution at room temperature for 1 h with moderate shaking, and then BCIP/NBT reagent was used (Cat# B1911; Sigma; USA). Pre-stained molecular mass markers were used to determine the molecular mass of matrix metalloproteinases (BioRad, Hercules, CA, USA). Representative blots were demonstrated.

### 2.5. Gelatinases’ Activity

Measurement of both MMPs’ actual specific activity was conducted in a microplate (Greiner Bio-One, Rainbach im Mühlkreis, Austria) which was previously coated using respective specific metalloproteinase antibodies (the same as in Western blot (WB) assays) [30]. The relevant sample of one hundred microliters was added to each well for the purpose of immobilization of the metalloproteinase. The microplate was incubated overnight at 4 °C. All redundant proteins were washed out using TBS-T buffer (50 mM Tris/HCl pH 7.4, 0.9% NaCl, 0.05% Tween 20). MMPs’ activity was measured in 100 μL of 50 mM Tris/HCl buffer, pH 7.5, containing 10 mM CaCl_2_, 150 mM NaCl, and 0.025% Brij 35 with 4 μM fluorogenic substrate (MCA-Pro-Leu-Ala-Cys(p-OMeBz)-Trp-Ala-Arg(Dpa)-H2)) (Cat#444258; Merck, Germany). The microplate was incubated at 37 °C for 1 h with gentle mixing. A total of 25 μL of 100 mM EDTANa_2_ was used to stop the reaction. The fluorogenic substrate degradation was measured by means of a multimode microplate reader (Tecan Infinite^®^ 200 PRO, Männedorf, Switzerland) with wavelengths of excitation at 325 nm and emission at 393 nm. Degraded substrate quantity was calculated on the basis of the calibration curve prepared under the same conditions with 7-amino-4-methylcoumarin (Sigma-Aldrich; Saint Louis, MO, USA). The specific activity of MMPs was given in katals per kg of protein.

### 2.6. Protein Determination

The protein concentration was measured using a method described by Bradford [31].

### 2.7. Statistical Analysis

Statistical analysis was performed using Statistica 10 (StatSoft Polska Sp. z o.o., Cracow, Poland). The results have been demonstrated as mean values and standard deviations (SD). MMPs’ content was expressed in nmol/g of fresh tissue. The MMPs’ activity was expressed in microkat/kg of protein. The statistical analysis was performed using the Student’s *t* test. We considered *p* values < 0.05 as statistically significant.

## 3. Results

**Figure 1:**
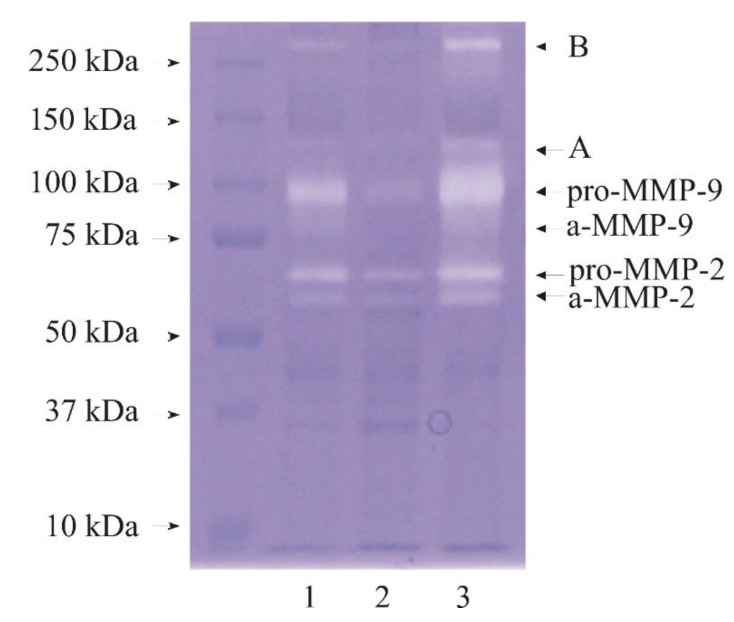
Zymogram shows the ability of gelatin degradation by proteins contained in the control tissue, low-, and high-grade bladder cancer samples. Twenty µg of protein in each sample was applied on the gel. Molecular mass standards were marked on the left of the zymogram. 1—control sample; 2—low-grade cancer sample; 3—high-grade cancer sample.

Representative zymogram is shown in Figure 1. Colorless bands present on the dark blue background show regions of gelatin degradation. They have been marked on with names of metalloproteinases or letters. Well-saturated band a-MMP-2 represents molecular weight of 60 kDa. It is present in every investigated tissue. It is the most intensive in the high-grade cancer tissue sample. The band marked pro-MMP-2 represents 65 kDa and is very similarly saturated to band a-MMP-2. These bands indicate the presence of MMP-2 in latent form (65 kDa) and in active form (60 kDa). The highest intensiveness of saturation of both forms is seen for HG urinary bladder cancer. Bands a-MMP-9 and pro-MMP-9 show the presence of enzymes in high molecular masses 80 kDa and 100 kDa, respectively. They represent active and latent form of MMP-9. Bands A and B are hard to analyze. It may indicate the presence of enzymes in higher molecular complexes (Figure 1).

### 3.1. MMP-2 and MMP-9 Content

**Figure 2:**
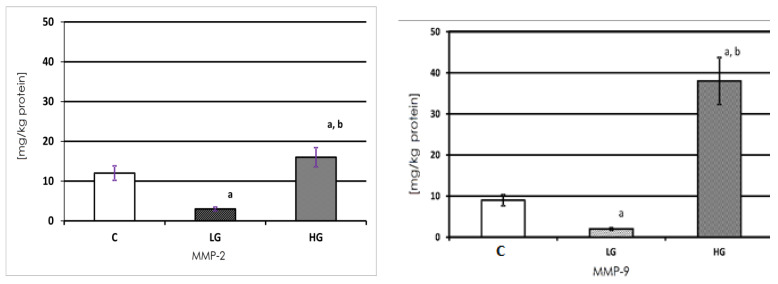
Content of MMP-2 and MMP-9 in control tissue and low-grade (LG) and high-grade (HG) urinary bladder cancer. a—*p* < 0.001 cancer vs. urinary bladder control; b—*p* < 0.001 high-grade vs. low-grade urinary bladder cancer.

Both gelatinases, expressed in milligrams per kg of protein, were present in both the healthy and cancerous tissue of the urinary bladder (Figure 2). MMP-2 present in the non-cancerous tissue extract showed 11.665 mg/kg of protein. The amount of that enzyme was significantly lower in LG urinary bladder cancer and higher in HG cancer. Nearly five times less of the enzyme was found in the LG tumor and approximately 30% more of MMP-2 was found in HG cancer in comparison with the control tissue.

The MMP-9 content was the highest in the HG tissue. The amount of MMP-9 was lower in LG cancer in comparison with the control and HG cancer. A normal urinary bladder wall contained 25% less of MMP-9 in comparison with the MMP-2 content in the same sample. A similar amount of MMP-9 was found in LG cancer and a more than two times higher amount was found in HG urinary bladder cancer in comparison with the MMP-2 content.

### 3.2. Western Blot Analysis of Investigated Gelatinases

The electrophoresis for WB analysis took place with disulfide bond reduction (reducing conditions) and without disulfide bond reduction (non-reducing conditions) using the same protein amount in every sample.

### 3.3. Expression of MMP-2 in Human Urinary Bladder

**Figure 3:**
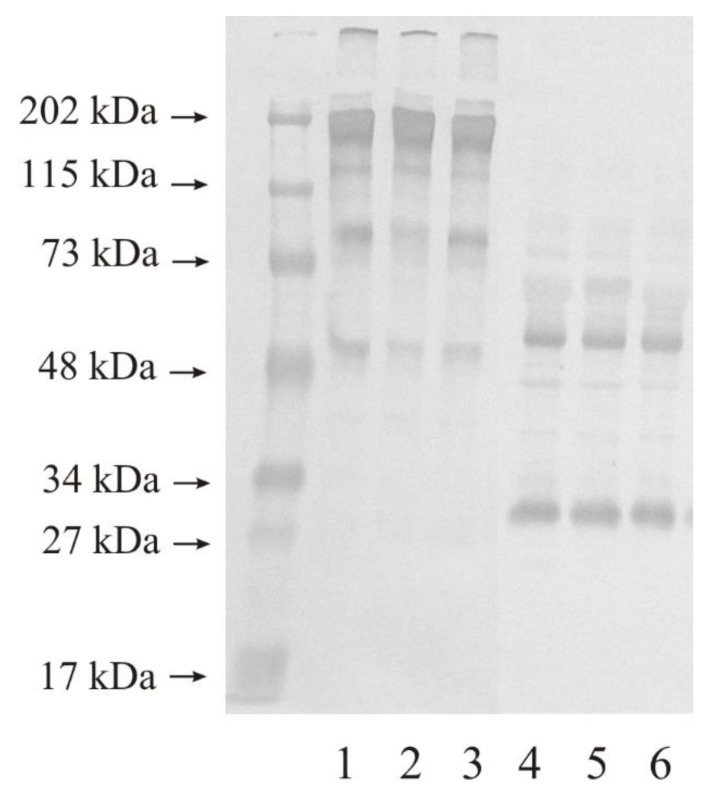
Western immunoblot of MMP-2 in control tissue and low-grade (LG) and high-grade (HG) urinary bladder cancer. An amount of 10 micrograms of protein, non-reducing conditions: lane 1—control urinary bladder, 2—low-grade bladder cancer, 3—high-grade bladder cancer; 10 micrograms of protein, reducing conditions: lane 4—control urinary bladder, 5—low-grade bladder cancer, 6—high-grade bladder cancer. It is a typical example of the WBs of the samples repeated five times.

Figure 3 presents the results of WB analysis of MMP-2 expression in the control urinary bladder and in tissues altered by the carcinogenetic process. Similar results were obtained for the WB measurements of the samples repeated five times. This figure shows 10 micrograms of protein on lane 1–3 (non-reducing conditions) and 4–6 (reducing conditions) of the same samples were used. In non-reducing conditions, normal urinary bladder had at least four bands with the molecular mass of 200 kDa, 120 kDa, 80 kDa, and 50 kDa (lane 1). The LG urinary bladder cancer tissue also had four bands with a molecular mass similar to the control sample (lane 2). The HG urinary bladder cancer tissue showed the same results as the control sample and the LG tissue (lane 3). In reducing conditions, the outcomes also revealed a reduced number of visible bands. All three kinds of samples showed two bands with a molecular mass of 50 kDa and 30 kDa (lane 4–6).

### 3.4. Expression of MMP-9 in Human Urinary Bladder

**Figure 4:**
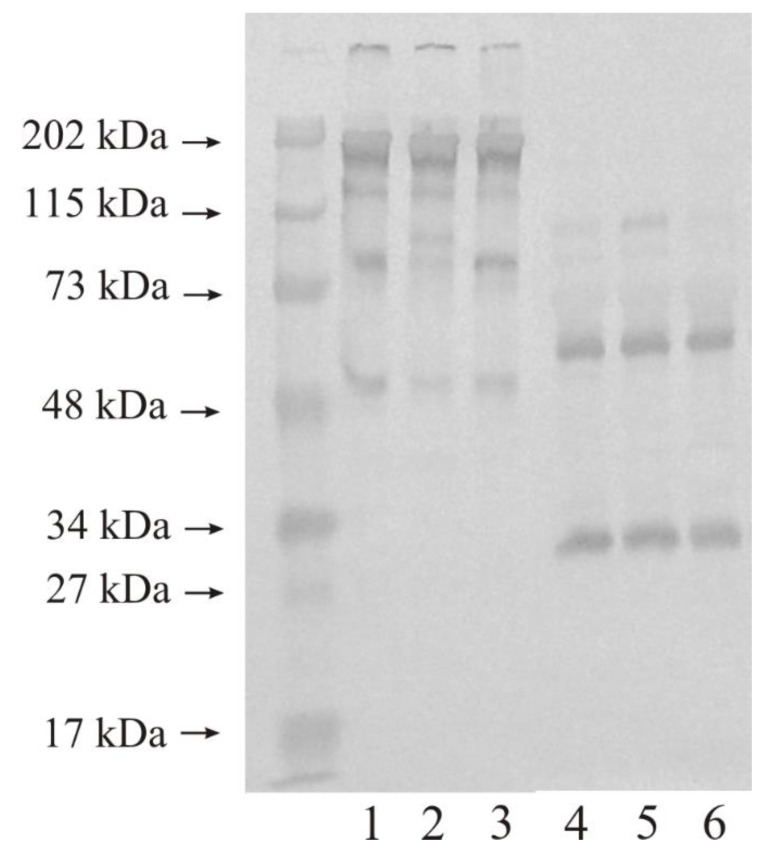
Western immunoblot of MMP-9 in control tissue and low-grade (LG) and high-grade (HG) urinary bladder cancer. A total of 10 micrograms of protein, non-reducing condition: lane 1—control urinary bladder, 2—low-grade bladder cancer, 3—high-grade bladder cancer; 10 micrograms of protein, reducing condition: lane 4—control urinary bladder, 5—low-grade bladder cancer, 6—high-grade bladder cancer. It is a typical example of the WBs of the samples repeated five times.

Figure 4 presents the results of WB analysis of MMP-9 expression in the control urinary bladder and in tissues altered by the carcinogenetic process. Similar results were obtained for the WB measurements of the samples repeated five times. A total of 10 micrograms of protein on lane 1–3 (non-reducing conditions) and 4–6 (reducing conditions) of the same samples were used. Normal urinary bladder had at least four bands with the following molecular mass: wide band of 195 kDa, and narrow bands of 120 kDa, 80 kDa, and 50 kDa (lane 1). The outcomes for LG urinary bladder cancer (lane 2) and HG urinary bladder cancer (lane 3) tissues were similar to the result for the control tissue except for a very light 80 kDa band in LG cancer. In reduction conditions, the number of visible bands was reduced to two only. The anti-MMP- antibody reacted with proteins with the molecular mass of approximately 55 kDa and 30 kDa (lane 4–6). The control tissue as well as the LG and HG cancerous tissues of the bladder revealed similar results visible in Figure 4.

### 3.5. Actual Activity of MMP-2 and MMP-9

Gelatinases actual activity was measured with the fluorometric method using oligopeptide as a substrate. Enzymes were isolated on a microplate pre-coated specifically for the gelatinase antibody, the same as was used for WB. The actual activity in the tissue extract was stated in katals per kg of total protein content.

The actual activity of MMP-2 was nearly 58 picokatals/kg of protein in the non-cancerous tissue of the urinary bladder. The LG urinary bladder cancer tissue demonstrated an increase in the actual activity of MMP-2. The increase in the grading of urinary bladder cancer was associated with the decrease in measured activity. The differences between low and high grades of cancer and control tissues were found to be significant.

**Figure 5:**
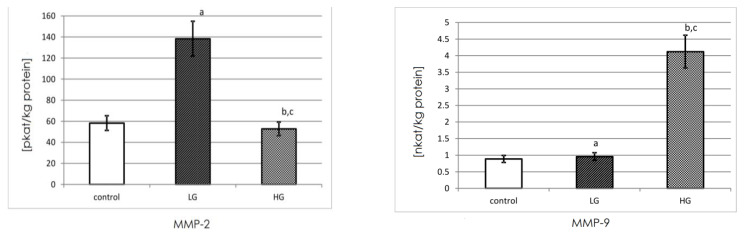
MMP-2 and MMP-9 actual activity in control tissue (*n* = 10) and low-grade (LG) (*n* = 10) and high-grade (HG) (*n* = 10) urinary bladder cancer. a—*p* < 0.001 low-grade cancer vs. urinary bladder control; b—*p* < 0.05 high-grade cancer vs. urinary bladder control; c—*p* < 0.001 high-grade vs. low-grade urinary bladder cancer.

The results demonstrated that the actual activity of MMP-9 was approximately 0.9 nanokatals per kg of total protein in control urinary bladder. A similar actual activity of MMP-9 was found in LG cancer tissues of the urinary bladder. The increase in the grading of cancer caused a huge increase in the actual activity of gelatinase-B by approximately four times. The actual activity of MMP-9 was found to be significantly different between both grades of cancer and control tissue (Figure 5).

### 3.6. Specific Activity of MMP-2 and MMP-9

The mean value of MMP-2 and MMP-9 specific activity was calculated and expressed in microkatals/kg of respective enzyme protein content in tissue extract.

The highest specific activity of MMP-2 was observed in the LG tissue of the urinary bladder. The calculated specific activity of MMP-2 decreased with an increase in cancer grading. The differences between low and high grades of cancer and control tissue were found to be significant.

**Figure 6:**
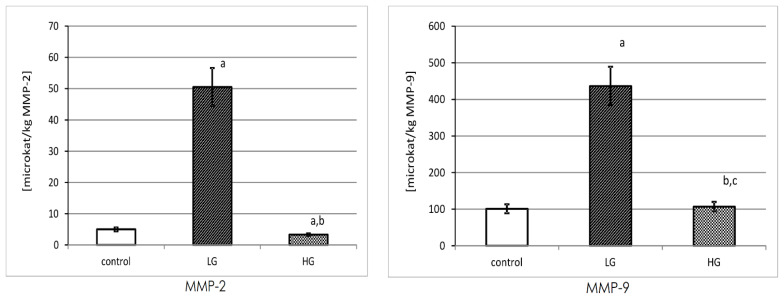
MMP-2 and MMP-9 specific activity in control tissue (*n* = 10) and low-grade (LG) (*n* = 10) and high-grade (HG) (*n* = 10) urinary bladder cancer. MMP-2: a—*p* < 0.001 cancer vs. urinary bladder control. b—*p* < 0.001 high-grade vs. low-grade urinary bladder cancer. MMP-9: a—*p* < 0.001 low grade-cancer vs. urinary bladder control. b—*p* < 0.05 high-grade cancer vs. urinary bladder control. c—*p* < 0.001 high-grade vs. low-grade urinary bladder cancer.

The results of MMP-9 specific activity were presented in microkatals/kg of enzyme. The non-cancerous tissue showed the lowest value of activity, being very similar to the high-grade tumor. The specific activity of MMP-9 was significantly increased in LG urinary bladder cancer. Moreover, it was found that the specific activity of this enzyme was approximately 4 times lower in the HG than in the LG cancer tissues (Figure 6).

## 4. Discussion

In this study, the bladder cancer tissues were examined to demonstrate the presence and activity of two gelatinases, MMP-2 and MMP-9.

The selection of this type of biological material was caused by only a few publications regarding the activity of gelatinases in the bladder cancer tissue. The vast majority of publications describe activity of these enzymes measured in serum or in the urine, but not in the tissue directly [3,32,33,34].

All pathological changes which take place in the body are best seen in the tissue where a particular change is taking place. Blood or urine reflects the state of the whole organism but not always a specific organ/tissue. Therefore, it was decided to take bladder tissue rather than urine or blood for study.

The expression of gelatinases in all examined tissues, determined by Western immunoblot was similar with the exception of MMP-9 in the low-grade cancer tissue of the urinary bladder. A very light and narrow band with a molecular mass of 80 kDa may constitute a free active form of MMP-9 [30]. Higher molecular mass bands showcased MMPs in complexes with other ECM proteins, inter alia TIMPs [11,35]. Many proteins of the ECM interact tentatively, but often with no impact on enzyme activity [11].

Performed zymogram analysis shows the presence of both enzymes in active and latent form in every investigated tissue. What should draw our attention is the highest intensiveness of saturation in the HG tissues which defines the highest collagenolytic activity in cancerous tissue of the urinary bladder. These results are consistent with the measurements performed by Kanda et al. [36], Wu et al. [37] and Papathoma et al. [38], where the levels of MMP-2 and MMP-9 showed statistically significant increase with tumor grade and invasiveness.

In the study, the content of examined metalloproteinases in all possible forms, i.e., latent, active, and TIMPs’- bound forms, in urinary bladder cancer was measured. Based on the results, it was found that the content of both gelatinases was the highest in HG and the lowest in LG tissues. However, in HG tissues the content of MMP-9 was significantly higher than MMP-2, where in LG and controls tissues it was similar.

Moreover, just a little higher (3 mg) content of MMP-2 in the healthy tissue than MMP-9 was found. This proves that both enzymes are present in a very similar amount in the healthy tissue, and both may be identically important in the reconstruction of ECM in the urinary bladder.

It has been observed that the actual activity of MMP- 9 is much higher than MMP-2 in all types of bladder tissues (nanokatals vs. picokatals/1 kg of total protein content) and is correlated with the grading of the cancer.

The actual activity of tested gelatinases in non-cancerous bladder tissue is different. For MMP-9, it was higher than MMP-2. Taking into account that the permanent process of ECM remodeling involves metalloproteinases, it seems that MMP-9 plays a significant role in maintaining the homeostasis of the extracellular matrix in healthy urinary bladder tissue.

Additionally, the specific activity of both gelatinases was measured. It was significantly higher for MMP-9 in comparison with MMP-2. Similar results were obtained by Davies at al. [39], who found that the activity of MMP-9 was approximately 10 times higher than the MMP-2. It appears that enzymatic activity is exerted to a greater extent by gelatinase B than gelatinase A. Moreover, it was found that the specific activities of both enzymes were highest for LG tissue and lowest for the control samples.

Based on the connection of the two measurements’ results, i.e., the content of all forms of the gelatinases with their specific activity in different tissues, it might be concluded that in the LG tissues both enzymes occur in the active form but in the HG tissues they occur in the latent or TIMPs- bounded forms.

### 4.1. Conclusions

We noticed changes in MMPs in healthy and cancerous tissues of the urinary bladder. The lowest content of both gelatinases was found in LG tumors tissues, but, in this tissue, the highest specific activity of both MMPs might be observed. It suggests that both enzymes occur more often in the active form in LG rather than in HG tissues, where the content of proteins is the highest but specific activity is lower.

Moreover, the observation connected with the results of all measurements performed for MMP-2 and MMP-9 in the tissues shows that MMP-9 is more involved in the growing and spreading of cancer cells in the bladder than MMP-2.

These observations may lead to perspective study connected with the searching of the expression and activation factors in bladder tissue, especially for MMP-9.

### 4.2. Limitations of the Study

The bladder tissues collected during surgery had to be frozen, and some part of each tissue was submitted for histopathological examination. The tissues were classified into a specific study group, low or high grade, after the receiving of the histopathological results. Unfortunately, not all of the taken patients’ tissues were suitable for qualification to a specific study group. This significantly increased the time of sample collection.

Another limit in our study was the difficulty of performing the measurements of the MMP-2 or MMP-9 activity. This was caused by the lack of a specific substrate for each of the MMPs. This issue was solved by the binding of MMP-2 or MMP-9 from the tested sample with a specific antibody immobilized on a fluorimetric plate. The remaining unbound MMPs from the sample were washed away. This provided the possibility of using a non-specific fluorimetric substrate that is degraded by all MMPs.

## Data Availability

The data that support the findings will be available on request under the corresponding author’s e-mail: jacek.kudelski@umb.edu.pl.
